# Short- and long-term haemodynamic consequences of transcatheter closure of atrial septal defect and patent foramen ovale

**DOI:** 10.1007/s12471-021-01543-0

**Published:** 2021-02-16

**Authors:** X. Jin, Y. M. Hummel, W. T. Tay, J. F. Nauta, N. S. S. Bamadhaj, J. P. van Melle, C. S. P. Lam, A. A. Voors, E. S. Hoendermis

**Affiliations:** 1 National Heart Centre Singapore, Singapore, Singapore; 2 Department of Cardiology, University of Groningen, University Medical Centre Groningen, Groningen, The Netherlands; 3 Duke-NUS Medical School, Singapore, Singapore

**Keywords:** ASD, HFpEF, Echocardiography, LVFP

## Abstract

**Background:**

Transcatheter atrial septal defect (ASD) and patent foramen ovale (PFO) closure might have opposite short- and long-term haemodynamic consequences compared with restricted interatrial shunt creation, which recently emerged as a potential treatment modality for patients with heart failure with preserved ejection fraction (HFpEF). Given the opposing approaches of ASD and PFO closure versus shunt creation, we investigated the early and sustained cardiac structural and functional changes following transcatheter ASD or PFO closure.

**Methods:**

In this retrospective study, adult secundum-type ASD and PFO patients with complete echocardiography examinations at baseline and at 1‑day and 1‑year follow-up who also underwent transcatheter closure between 2013 and 2017 at the University Medical Centre Groningen, the Netherlands were included.

**Results:**

Thirty-nine patients (mean age 48 ± standard deviation 16 years, 61.5% women) were included. Transcatheter ASD/PFO closure resulted in an early and persistent decrease in right ventricular systolic and diastolic function. Additionally, transcatheter ASD/PFO closure resulted in an early and sustained favourable response of left ventricular (LV) systolic function, but also in deterioration of LV diastolic function with an increase in LV filling pressure (LVFP), as assessed by echocardiography. Age (β = 0.31, *p* = 0.009) and atrial fibrillation (AF; β = 0.24, *p* = 0.03) were associated with a sustained increase in LVFP after transcatheter ASD/PFO closure estimated by mean E/e’ ratio (i.e. ratio of mitral peak velocity of early filling to diastolic mitral annular velocity). In subgroup analysis, this was similar for ASD and PFO closure.

**Conclusion:**

Older patients and patients with AF were predisposed to sustained increases in left-sided filling pressures resembling HFpEF following ASD or PFO closure. Consequently, these findings support the current concept that creating a restricted interatrial shunt might be beneficial, particularly in elderly HFpEF patients with AF.

**Supplementary Information:**

The online version of this article (10.1007/s12471-021-01543-0) contains supplementary material, which is available to authorized users.

## Introduction

Atrial septal defect (ASD) is one of the most common adult congenital heart diseases, and the chronic left-to-right atrial shunt might eventually lead to the development of pulmonary arterial hypertension, arrhythmia and heart failure [1]. ASD closure is indicated at all ages, and closure of small ASDs and PFO can be indicated in cases of cryptogenic strokes or in divers with decompression illness [2]. However, older age at closure, male sex, comorbidities such as hypertension, diabetes mellitus and atrial fibrillation (AF), and larger ASDs may predispose to worse outcomes despite ASD closure [3–7].

Recently, the artificial creation of a restricted interatrial shunt with a transcatheter device has emerged as a potential treatment modality for patients with heart failure with preserved ejection fraction (HFpEF) by means of reducing left atrial (LA) pressure [8]. Given the opposing approaches of ASD/PFO closure versus creation, as well as overlapping risk factors for HFpEF and worse prognosis of transcatheter ASD closure, we hypothesised that some patients undergoing ASD/PFO closure may display haemodynamic characteristics resembling HFpEF. We aimed to test this hypothesis by investigating the early and sustained cardiac structural and functional changes following transcatheter ASD/PFO closure using sequential echocardiograms and their clinical correlates.

## Methods

**Fig. 1:**
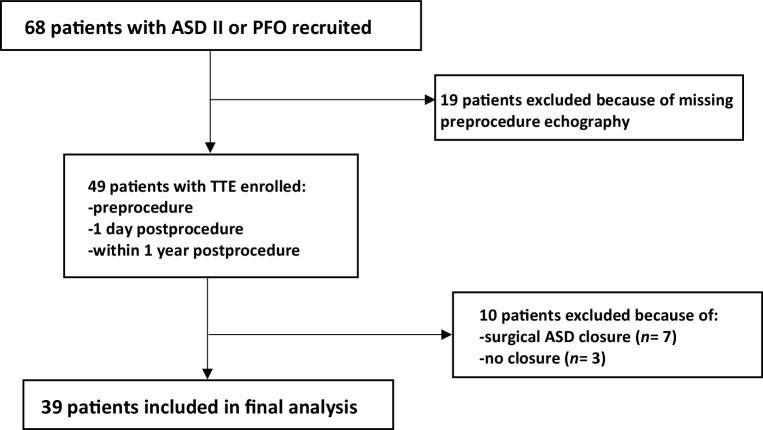
Flowchart illustrating enrolment process of study participants. *ASD II* secundum-type atrial septal defect, *PFO* patent foramen ovale, *TTE* transthoracic echocardiography

This retrospective study recruited adult patients (age > 18 years) who underwent transcatheter ASD/PFO closure with comprehensive echocardiographic datasets between 2013 and 2017 at the University Medical Centre Groningen, the Netherlands. Transcatheter ASD/PFO closure was indicated following the guidelines of the European Society of Cardiology (ESC) and the Netherlands Society of Cardiology [9]. Among the 68 recruited patients 39 patients with transcatheter ASD/PFO closure were included in the present analyses after excluding 29 patients due to unavailability of baseline echocardiography (*n* = 19), surgical approach transition (*n* = 7) or technical reasons (*n* = 3). The study flow chart is presented in Fig. 1. Of the 39 patients, 28 had a large ASD, and 11 had an ASD < 1 cm or a PFO, both with cryptogenic strokes as indication for closure. This study was conducted in accordance with local and national laws and regulations regarding retrospective research on clinical data, as verified and confirmed by the institutional review board of the University Medical Centre Groningen.

Transcatheter ASD closure was performed using the Amplatzer Septal Occluder or PFO occluder (St. Jude Medical, St. Paul, MN, USA) or the Figulla ASD Occluder or PFO occluder (Occlutech GmbH, Jena, Germany), under general anaesthesia and guidance of intraprocedural transoesophageal echocardiography. Before closure, mean right atrial (RA) pressure, mean LA pressure, mean pulmonary arterial pressure, pulmonary capillary wedge pressure and ratio of total pulmonary blood flow to total systemic blood flow (Qp/Qs ratio) were obtained using a Swan-Ganz catheter, and all measurements were recorded at end expiration based on average of five cardiac cycles.

Comprehensive transthoracic echocardiography (GE Vivid‑7 or Vivid-E9, General Electric, Horten, Norway) and all offline measurements including two-dimensional speckle-tracking echocardiography analysis were performed following the ESC guideline [10], at baseline, at 1‑day follow-up and within 1 year of follow-up, respectively. Right ventricular (RV) global longitudinal strain (GLS), RA reservoir GLS, left ventricular (LV) GLS and LA reservoir GLS were all measured based on recommended guideline [10]. RV systolic function parameters included RVGLS, systolic tissue velocity of the lateral tricuspid annulus, tricuspid annular plane systolic excursion, and RV fractional area change. RV diastolic function parameters included RA volume index, RA reservoir GLS, diastolic tissue velocity of the lateral tricuspid annulus, and RV isovolumic relaxation time.

Pulmonary artery systolic pressure was estimated using Bernoulli’s equation of peak tricuspid regurgitant velocity and RA pressure estimation, based on the size and collapsibility of the inferior vena cava [10]. LV systolic function parameters were LV ejection fraction (LVEF), which was assessed by biplane method, and LVGLS. LV diastolic function parameters were mitral inflow, annular velocity and transmitral to averaged septal and lateral annular early diastolic velocity ratio (E/e’ ratio), LA volume index and LA reservoir GLS.

### Statistical analysis

Continuous variables are presented as mean ± standard deviation (SD) or as median with range. Categorical variables are expressed as frequency and percentage. Differences in echocardiographic characteristics between baseline versus 1‑day or 1‑year follow-up were compared using a paired *t*-test.

A linear regression model was used to evaluate the association between clinical risk factors of HFpEF and cardiac functional changes assessed by echocardiography. A multivariable model was created by including all variables in the univariable analysis. Stratified analysis was performed for the significant echocardiographic changes at 1‑day and 1‑year follow-up after transcatheter closure by ASD versus PFO. *P* values ≤ 0.05 were considered statistically significant.

All data analyses were performed using SPSS, version 22.0 (SPSS, Chicago, IL, USA).

## Results

The mean age of patients included (*n* = 39) was 48 ± 16 years, and 61.5% were women. Among all patients, 28 (72%) had a large ASD with RV volume overload, 12 (30.7%) had a history of hypertension, 9 (23.7%) had a history of AF and 2 (5.1%) had a history of diabetes mellitus.

Tab. 1 summarises demographic characteristics and invasive haemodynamic data at baseline in ASD and PFO patients. Median ASD and PFO size with invasive balloon sizing was 17.1 and 11.8 mm, respectively. Median device size for ASD (waist) and PFO (RA disc) was 20 mm and 28.7 mm, respectively. Mean Qp/Qs ratio was 2.0 in patients with ASD. Mean LA pressure was 7.1 mm Hg in ASD patients and 6.8 mm Hg in PFO patients. Mean pulmonary arterial pressure was 16.9 versus 13.4 mm Hg in ASD and PFO patients, respectively. There were no major complications of the procedures, and none of the patients showed signs of pulmonary hypertension within 1‑year echocardiography follow-up. The mean follow-up time was 8 ± 3 months.

**Table 1:** Clinical and invasive haemodynamic characteristics at baseline in patients with ASD or PFO

Variable	ASD (*n* = 28)	PFO (*n* = 11)
Age, years	48 ± 16	45 ± 17
Women	15 (53.5)	9 (81.8)
BMI, kg/m^2^	26.9 ± 4.1	24.1 ± 3.2
Hypertension	9 (32.1)	3 (27.7)
Hyperlipidaemia	3 (10.7)	1 (9.1)
Diabetes mellitus	2 (7.1)	0
Atrial fibrillation	7 (25)	2 (18.1)
Coronary artery disease	3 (10.7)	0
ASD size with balloon sizing, mm	17.1 (7–25)	NA
ASD device size (waist), mm	20 (10–35)	NA
PFO size with balloon sizing, mm	NA	11.8 (5–19)
PFO device size (right atrial disc), mm	NA	28.7(25–31)
Qp/Qs ratio	2.0 ± 0.6	NA
Mean pulmonary arterial pressure, mm Hg	16.9 ± 4.3	13.4 ± 5.0
Mean left atrial pressure, mm Hg	7.1 ± 2.4	6.8 ± 2.1
Mean right atrial pressure, mm Hg	6.4 ± 2.6	3.6 ± 1.7

Data are *n* (%), mean ± standard deviation, or median (range)

*ASD* atrial septal defect, *PFO* patent foramen ovale, *BMI* body mass index,* Qp/Qs ratio* ratio of total pulmonary blood flow to total systemic blood flow, *NA* not applicable

**Fig. 2:**
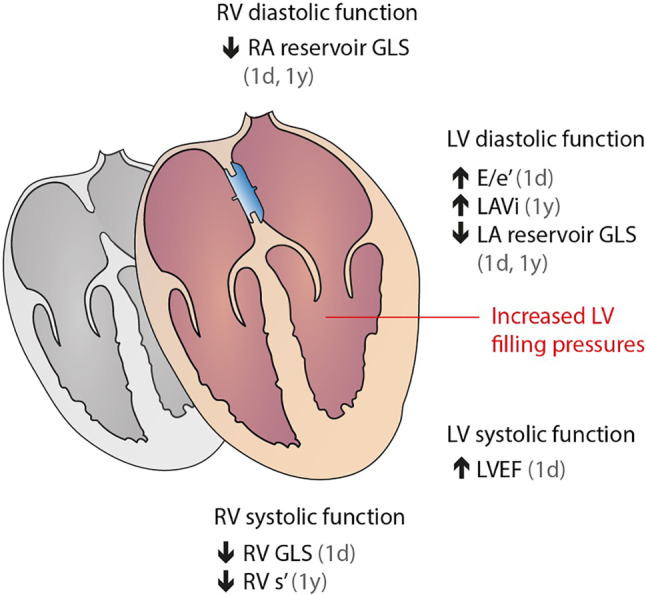
Early and sustained cardiac remodelling and functional changes following transcatheter atrial septal defect or patent foramen ovale closure. *RV* right ventricular, *RA* right atrial, *LV* left ventricular, *LA* left atrial,* GLS* global longitudinal strain, *E/e’* *ratio* of mitral peak velocity of early filling to diastolic mitral annular velocity, *LAVi* left ventricular volume index, *LVEF* left ventricular ejection fraction, *RV s’* systolic tissue velocity of the lateral tricuspid annulus

Tab. 2 demonstrates the longitudinal changes of echocardiographic parameters (mean ± SD) following transcatheter ASD/PFO closure in all included patients. Notably, transcatheter ASD/PFO closure resulted in an early and sustained reduction of RV dimensions, an early increase in LV systolic function and a stepwise increase in LV dimensions/mass. Transcatheter ASD/PFO closure resulted in an early and persistent decrease in RV systolic and diastolic function and in deterioration of LV diastolic function. Following transcatheter ASD/PFO closure, there were no significant early or sustained changes in pulmonary artery systolic pressure. Fig. 2 summarises the early and sustained cardiac remodelling and functional changes following transcatheter ASD/PFO closure we found.

**Table 2:** Echocardiographic characteristics at baseline, at day 1 and within 1 year following transcatheter ASD/PFO closure

Variable	Baseline	Day 1	Within 1 year	*P*_interaction for_ _ASD vs PFO_
*Right ventricular systolic function*				
RVGLS, %	−20.7 ± 3.1	−18.5 ± 4.6^a^	−17.9 ± 6.9	–
RV TDI s’, cm/s	14.3 ± 2.3	13.4 ± 2.1	12.7 ± 2.8^b^	0.30
TAPSE, mm	24.4 ± 5.6	23.7 ± 4.7	23.1 ± 4.5	0.36
RVFAC, %	40.4 ± 7.5	41.5 ± 6.8	39.7 ± 6.7	–
*Right ventricular diastolic function*				
RV-IVRT, ms	61.8 ± 21.9	61.8 ± 18.8	72.8 ± 23.6^b^	0.26
RAVi, mL/m^2^	58.7 ± 35.8	47.4 ± 25.9^a^	44.1 ± 19.7^b^	0.44
RA reservoir GLS, %	36.9 ± 14.6	31.4 ± 13.2^a^	30.4 ± 13.4^b^	0.42
*Left ventricular systolic function*				
LVEF (biplane), %	54.0 ± 7.0	56.7 ± 8.6^a^	55.1 ± 7.3	0.88
Stroke volume, mL	54.0 ± 7.0	56.7 ± 8.6	58.03 ± 16.3	–
LVGLS, %	−15.7 ± 3.1	−15.8 ± 2.9	−16.0 ± 3.2	0.31
*Left ventricular diastolic function*				
MV E/A	1.4 ± 0.8	1.5 ± 1.3	1.4 ± 0.5	–
E/e’ ratio	6.3 ± 2.0	7.3 ± 3.2^a^	6.8 ± 1.8	0.76
LAVi, mL/m^2^	29.6 ± 12.4	29.9 ± 11.2	34.5 ± 13.9^b^	0.89
LA reservoir GLS, %	32.8 ± 13.9	26.7 ± 9.9^a^	26.7 ± 10.7^b^	0.79
*Cardiac dimensions and Doppler parameters*				
LVIDd, mm	48.0 ± 5.7	48.3 ± 5.0	50.0 ± 4.5^b^	–
LVIDs, mm	34.3 ± 5.7	33.9 ± 4.9	35.1 ± 5.0	–
LVMi, g/m^2^	72.9 ± 16.9	74.4 ± 14.7	81.7 ± 17.1^b^	–
RV basal diameter, mm	40.5 ± 8.6	37.9 ± 7.8^a^	37.6 ± 6.9^b^	–
LA area, cm^2^	21.8 ± 18.0	18.9 ± 3.57	20.5 ± 4.9	–
RA area, cm^2^	18.3 ± 6.0	16.7 ± 5.8^a^	16.0 ± 4.7^b^	–
TR velocity, m/s	2.1 ± 0.8	2.3 ± 0.8	2.2 ± 0.7	–
PASP, mm Hg	20.7 ± 12.3	23.4 ± 15.1	21.6 ± 11.7	–

Data are mean ± standard deviation

*T‑ASD* transcatheter atrial septal defect, *PFO* patent foramen ovale, *RVGLS* right ventricular global longitudinal strain, *RV TDI s’* peak systolic velocity of tricuspid annulus by tissue Doppler imaging, *TAPSE* tricuspid annular plane systolic excursion, *RVFAC* right ventricular fractional area change, *RV-IVRT* right ventricular isovolumic relaxation time, *RAVi* right atrial volume index, *GLS* global longitudinal strain, *LVEF* left ventricular ejection fraction, *LVGLS* left ventricular global longitudinal strain, *MV E/A* mitral valve E velocity divided by A velocity, *E/e’ ratio* ratio of mitral peak velocity of early filling to diastolic mitral annular velocity, *LAVi* left ventricular volume index, *LA* left atrial, *LVIDd* left ventricular internal diameter in diastole, *LVIDs* left ventricular internal diameter in systole, *LVMi* left ventricular mass index, *TR velocity* tricuspid regurgitant peak velocity, *PASP* pulmonary artery systolic pressure

^a^ *P* < 0.05 between baseline and 1‑day follow-up

^b^ *P* < 0.05 between baseline and 1‑year follow-up

Following transcatheter ASD/PFO closure, RV basal diameter was significantly reduced at 1‑day follow-up (37.9 ± 7.8 vs 40.5 ± 8.6 mm at baseline, *p* = 0.01), a change that persisted at 1‑year follow-up (37.6 ± 6.9 mm, *p* = 0.04 for difference with baseline). Additionally, RA volume index showed a significant reduction at 1‑day follow-up (47.4 ± 25.9 vs 58.7 ± 35.8 mL/m^2^ at baseline, *p* = 0.04) and at 1‑year follow-up (44.1 ± 19.7 mL/m^2^, *p* = 0.02 for difference with baseline).

Irrespective of reduction in RV or RA dimension due to shunt closure, transcatheter ASD/PFO closure resulted in an early and continuous decrease in RV systolic and diastolic function. As for RV systolic functional assessments, RVGLS showed a significant decrease in the absolute value at 1‑day follow-up (−18.5 ± 4.6 vs −20.6 ± 3.1%, *p* = 0.01), and this trend remained at 1‑year follow-up (−17.9 ± 6.9%, *p* = 0.05 for difference with baseline). Systolic tissue velocity of the lateral tricuspid annulus also showed a significant and sustained decrease at 1‑year follow-up compared with baseline (12.7 ± 2.8 vs 14.3 ± 2.3 cm/s, *p* = 0.003). RA reservoir GLS (as a measurement of RV diastolic function) was significantly decreased at 1‑day follow-up (31.4 ± 13.2 vs 36.9 ± 14.6%, *p* = 0.02) and remained significantly lowered at 1‑year follow-up (30.4 ± 13.4%, *p* = 0.03 for difference with baseline). Moreover, RV isovolumic relaxation time also showed a significant increase within 1 year after transcatheter ASD/PFO closure compared with baseline, in all suggesting a sustained decline in RV diastolic function after closure.

LV systolic function showed early improvement (LVEF 54.0 ± 7.0 at baseline vs 56.7 ± 8.6 at 1‑day follow-up, *p* = 0.04). We also found a stepwise increase between baseline and at 1 year in LV internal diameter in diastole (from 48.0 ± 5.7 to 50.0 ± 4.5 mm, *p* = 0.008) and in LV mass index (from 72.9 ± 16.9 g to 81.7 ± 17.1 g/m^2^, *p* < 0.001).

LVFP showed an early and sustained increase. Mean E/e’ ratio significantly increased from 6.3 ±2.0 cm/s at baseline to 7.3 ± 3.2 at 1 day (*p* = 0.003), and LA volume index significantly increased from 29.6 ± 12.4 mL/m^2^ at baseline to 34.5 ± 13.9 mL/m^2^ at 1 year (*p* = 0.04). LA reservoir GLS significantly decreased from 32.8 ± 13.9% at baseline to 26.7 ± 9.9% at 1 day (*p* = 0.006) and to 26.7 ± 10.7% at 1 year (*p* = 0.01).

In stratified analyses by large ASD versus small ASD/PFO (Tab. 2), cardiac structural and functional changes, including left- and right-sided systolic and diastolic function, after transcatheter closure at 1‑day and 1‑year follow-up were directionally similar in both groups. Tabs. 3 and 4 in the Electronic Supplementary Material demonstrate longitudinal changes of echocardiographic parameters following transcatheter ASD and PFO closure, respectively. Changes in LA reservoir GLS, RA reservoir GLS and systolic tissue velocity of the lateral tricuspid annulus did not reach statistical significance in patients with small ASD/PFO at 1‑year follow-up after transcatheter closure, likely due to the small sample size.

Furthermore, the presence of AF at enrolment and ASD size were significantly associated with sustained changes of LA volume index (AF: β = 0.52, *p* = 0.005; ASD size: β = 0.48, *p* = 0.01) and LA reservoir GLS (AF: β = −0.48, *p* < 0.01; ASD size: β = −0.95, *p* < 0.01) in univariable analysis. Notably, age (β = 0.31, *p* = 0.009) and AF (β = 0.24, *p* = 0.03) remained significantly associated with sustained changes of E/e’ ratio in multivariable models after adjusting for baseline mean E/e’ ratio, sex, hypertension and ASD size (Tab. 5 in Electronic Supplementary Material).

## Discussion

In this study, we demonstrated early and sustained cardiac remodelling and functional changes following transcatheter ASD/PFO closure. In terms of structural changes, transcatheter ASD/PFO closure resulted in decreased RV and RA sizes and increased LV and LA sizes. In terms of functional changes, we found an acute and persistent decline in right heart function, with a parallel decline in both RV systolic and diastolic function. On the left side, transcatheter ASD/PFO closure resulted in early and sustained improvement in LV systolic function, but also in deterioration of LV diastolic function with an increase in echo-estimated LV filling pressure. The continuous increase in LV filling pressure was associated with older age and presence of AF, but not with ASD size, ASD versus PFO, sex or hypertension.

Focusing on right heart changes following transcatheter ASD/PFO closure, our results are consistent with those of some, but not all prior studies [4, 11–14]. Similar to our findings, Stephensen et al. saw an acute decline in RV ejection fraction (RVEF) at day 1 following transcatheter ASD closure, which remained a decreasing trend till 12 months of follow-up [12]. On the contrary, Schoen et al. found improvement in RVEF by MRI in association with a reduction in RV systolic pressure [13]. We postulate that these divergent results are due to differences in study populations with respect to RV afterload. ASD closure is expected to normalise RV volume overload and thereby relieve the RV from its compensatory increase in RVEF to cope with the additional shunt volume.

In the absence of significant pulmonary hypertension, a reduction in RV volume with ASD closure may therefore be associated with a reduction in RVEF or RV systolic strain, albeit still within the normal range, as observed in our study and others [11]. Interestingly, in our study, RV functional changes after transcatheter closure were similar in ASD patients (with RV volume load) and PFO patients (without RV volume load) after subgroup analysis, suggesting RV adaptation following transcatheter ASD/PFO closure is a complicated process that is affected by more than volume overload and pressure afterload.

Conversely, from the standpoint of interatrial shunt creation in HFpEF, although it is as restrictive as a PFO, there is concern that the additional RV volume from the shunt may tip the right heart into failure, particularly in the presence of pulmonary hypertension from HFpEF itself. Furthermore, LV underfilling may occur following interatrial shunting from left to right. Notably, recent studies of interatrial shunt device in HFpEF have not reported these adverse effects, although it is important to bear in mind the strict inclusion and exclusion criteria applied in these trials (excluding significant pulmonary hypertension or RV failure), the restricted size of the iatrogenic PFO and the limited follow-up of patients receiving this relatively new treatment [8, 15–20].

With regard to left-sided filling pressures, our findings regarding changes in LV diastolic function following ASD closure are consistent with those of prior studies. Ewert et al. found that transcatheter ASD closure in the elderly is associated with an increase in LVFP and subsequent congestive heart failure due to masked underlying LV diastolic dysfunction [21, 22]. We extended the prior data by including echo-tissue Doppler and strain measurements of LV and LA function. The E/e’ ratio is a widely accepted surrogate for LVFP estimation and proved to be associated with both haemodynamics and prognosis in patients with HFpEF [23, 24].

Likewise, LA reservoir GLS, as an additional reflection of LA myocardial compliance, emerged as a promising noninvasive surrogate for LVFP estimation [25]. Prior studies have shown an acute increase in LVFP, estimated as E/e’ ratio [26, 27], which is consistent with our results. Furthermore, the current study, in which we used a combination of mean E/e’ ratio, LA volume index and LA reservoir GLS, demonstrated that transcatheter ASD/PFO closure resulted in both an acute and sustained increase in LVFP. In addition, we found that older age and the presence of AF were independently related to sustained increases in LVFP. The important influence of age at ASD closure on changes in LVFP has been shown before; however, the influence of AF has not been extensively studied to date.

### Limitations

The major limitation of our study is the relatively small sample size, although we included consecutive patients with ASD and PFO undergoing transcatheter ASD closure with full echocardiographic datasets of diastolic function up to 1 year. The age difference between the current population and much elderly HFpEF patients is also a limitation to be speculated about. The current study results might underestimate iatrogenic ASD creation in much elderly HFpEF patients. We acknowledge that absolute changes were small and of uncertain clinical significance. Nonetheless, this is one of the first studies to comprehensively assess patients with strain echocardiography before and after ASD closure, and our findings may carry implications for the propensity to develop HFpEF after ASD or PFO closure or, conversely, the propensity to benefit from iatrogenic ASD creation in HFpEF.

## Conclusion

Older patients and AF patients are predisposed to sustained increases in left-sided filling pressures following ASD or PFO closure. These findings support the concept that elderly HFpEF patients with AF in particular might benefit from the artificial creation of an atrial left-to-right shunt.

### What’s new?

- In this study, transcatheter closure of an atrial septal defect (ASD) or a patent foramen ovale (PFO) resulted in an early and sustained increase in left ventricular (LV) filling pressure, as assessed by echocardiography.
- Older patients with ASD or PFO and patients with atrial fibrillation were predisposed to sustained increases in LV filling pressure resembling heart failure with preserved ejection fraction (HFpEF) following interatrial shunt closure.
- The findings of the present study support the current concept that creating a restricted interatrial shunt in patients with HFpEF might result in acute and sustained improvement in LV diastolic dysfunction and a decrease in LV filling pressures.

## Supplementary Information

Supplementary Table 3: Echocardiographic characteristics at baseline, day 1 and 1 year after following ASD closure

Supplementary Table 4: Echocardiographic characteristics at baseline, day 1 and 1 year after following PFO closure

Supplementary Table 5: Independent predictors of sustained changes in echocardiographic estimations of left ventricular filling pressures. after T‑ASD/PFO closure
