# Supplementary material for: Short- and long-term haemodynamic consequences of transcatheter closure of atrial septal defect and patent foramen ovale
Source: Neth Heart J. 2021 Feb 16;29(7-8):402–8. doi: 10.1007/s12471-021-01543-0 (PMC8271075; doi:10.1007/s12471-021-01543-0)
Supplement: Supplementary file 1 — Supplementary Table 3: Echocardiographic characteristics at baseline, day 1 and 1 year after following ASD closure [file 12471_2021_1543_MOESM1_ESM.docx]

**Supplementary Table 3: Echocardiographic characteristics at baseline, day 1 and 1 year after following ASD closure**

|  | Baseline | Day 1 | Within 1 year |
| --- | --- | --- | --- |
| RVGLS, % | -20.5±2.9 | -17.8±4.6* | -17.0±8.0 |
| RV TDI s', cm/s | 14.6±2.4 | 13.3±2.0 | 12.8±2.9** |
| TAPSE, mm | 24.8±6.2 | 23.7±4.4 | 23.1±4.8 |
| RVFAC, % | 41.6±5.9 | 41.3±7.3 | 40.4±6.5 |
| RV-IVRT, ms | 66.0±20.2 | 62.7±21.5 | 77.6±25.3** |
| RAVi, ml/m2 | 63.2±38.8 | 48.2±23.1* | 46.4±18.6** |
| RA reservoir GLS, % | 36.2±14.2 | 30.4±12.7* | 31.2±13.7** |
| LVEF (Biplane, %) | 51.6±11.7 | 56.4±8.9* | 54.7±7.3 |
| Stroke Volume, ml | 61.5±17.1 | 58.5±10.5 | 62.3±14.5 |
| LVGLS, % | -15.8±4.1 | -16.6±3.0 | -17.4±2.5 |
| MV E/A | 1.3±0.7 | 1.5±1.6 | 1.3±0.5 |
| Mean E/e' | 6.9±2.6 | 7.9±2.8* | 6.9±2.0 |
| LAVi, ml/m2 | 30.7±10.4 | 31.2±11.3 | 36.4±14.5** |
| LA reservoir GLS, % | 30.8±13.3 | 26.8±10.0* | 26.1±11.7** |
| LVIDd, mm | 49.0±5.6 | 49.3±4.5 | 50.6±4.5** |
| LVIDs, mm | 34.9±5.7 | 34.8±4.9 | 36.0±5.3 |
| LVMi, g/m2 | 75.9±17.8 | 77.8±13.4 | 85.4±17.2** |
| RV base diameter, mm | 41.9±8.1 | 38.9±7.0* | 40.0±6.6** |
| LA area, cm^2^ | 21.8±18.0 | 18.9±3.57 | 20.5±4.9 |
| RA area, cm^2^ | 18.9±5.8 | 17.3±5.3* | 16.7±4.2** |
| TR velocity, m/s | 2.2±0.8 | 2.4±0.8 | 2.3±0.7 |
| PASP, mmHg | 21.7±12.1 | 25.4±15.5 | 22.9±11.6 |
