# Supplementary material for: Short- and long-term haemodynamic consequences of transcatheter closure of atrial septal defect and patent foramen ovale
Source: Neth Heart J. 2021 Feb 16;29(7-8):402–8. doi: 10.1007/s12471-021-01543-0 (PMC8271075; doi:10.1007/s12471-021-01543-0)
Supplement: Supplementary file 2 — Supplementary Table 4: Echocardiographic characteristics at baseline, day 1 and 1 year after following PFO closure [file 12471_2021_1543_MOESM2_ESM.docx]

**Supplementary Table 4: Echocardiographic characteristics at baseline, day 1 and 1 year after following PFO closure**

|  | Baseline | Day 1 | Within 1 year |
| --- | --- | --- | --- |
| RVGLS, % | -20.7±3.6 | -19.9±4.2 | -19.9±3.1 |
| RV TDI s', cm/s | 13.4±2.1 | 13.5±2.5 | 12.7±1.0 |
| TAPSE, mm | 23.0±3.8 | 23.6±5.3 | 23.5±3.3 |
| RVFAC, % | 40.4±7.5 | 41.5±6.8 | 39.7±6.7 |
| RV-IVRT, ms | 61.8±21.9 | 61.8±18.8 | 72.8±23.6** |
| RAVi, ml/m2 | 58.7±35.8 | 47.4±25.9* | 44.1±19.7** |
| RA reservoir GLS, % | 36.9±14.6 | 31.4±13.2 | 30.4±13.4 |
| LVEF (Biplane, %) | 53.7±8.3 | 57.4±8.3* | 56.0±7.9 |
| Stroke Volume, ml | 49.0±8.7 | 58.4±18.1 | 52.1±15.6 |
| LVGLS, % | -15.8±4.1 | -16.7±3.1 | -17.4±2.6 |
| MV E/A | 1.4±0.6 | 1.4±0.3 | 1.5±0.6 |
| Mean E/e' | 6.0±1.8 | 6.9±2.9* | 6.5±1.6 |
| LAVi, ml/m2 | 24.3±16.5 | 26.1±10.3 | 30.7±12.1** |
| LA reservoir GLS, % | 36.1±14.9 | 26.4±10.3* | 28.2±8.5 |
| LVIDd, mm | 45.2±5.4 | 46.3±5.8 | 48.4±4.7** |
| LVIDs, mm | 32.2±5.6 | 31.8±4.6 | 32.5±3.7 |
| LVMi, g/m2 | 64.2±11.9 | 66.2±14.8 | 73.2±14.9** |
| RV base diameter, mm | 36.0±8.8 | 35.4±9.3 | 32.0±4.3** |
| LA area, cm^2^ | 21.8±18.0 | 18.9±3.57 | 20.5±4.9 |
| RA area, cm^2^ | 16.7±6.8 | 15.5±5.9* | 14.5±5.8** |
| TR velocity, m/s | 1.9±0.8 | 2.3±0.9 | 2.2±0.7 |
| PASP, mmHg | 17.9±13.7 | 24.3±15.6 | 20.8±11.3 |
