# Supplementary material for: Short- and long-term haemodynamic consequences of transcatheter closure of atrial septal defect and patent foramen ovale
Source: Neth Heart J. 2021 Feb 16;29(7-8):402–8. doi: 10.1007/s12471-021-01543-0 (PMC8271075; doi:10.1007/s12471-021-01543-0)
Supplement: Supplementary file 3 — Supplementary Table 5: Independent predictors of sustained changes in echocardiographic estimations of left ventricular filling pressures. after T‑ASD/PFO closure [file 12471_2021_1543_MOESM3_ESM.docx]

**Supplementary Table 3: Independent predictors of sustained changes in echocardiographic estimations of left ventricular filling pressures. after T-ASD/PFO closure**

| **Clinical co-morbidities** | **Delta E/e’** | | | | **Delta LAVi** | | | | **Delta LA reservoir GLS** | | | | |
| --- | --- | --- | --- | --- | --- | --- | --- | --- | --- | --- | --- | --- | --- |
|  | Univariate | | Multi-variate | | Univariate | | Multi-variate | | Univariate | | Multi-variate | | |
|  | βCoefficient | P value | βCoefficient | P value | βCoefficient | P value | βCoefficient | P value | βCoefficient | P value | βCoefficient | P value |  |
| **Age** | 0.47 | <0.001 | 0.31 | 0.009 | 0.35 | 0.07 | - | - | -0.21 | 0.29 | - | - |  |
| **Sex** | 0.22 | 0.12 | - | - | 0.12 | 0.51 | - | - | -0.20 | 0.16 | - | - |  |
| **HT** | -0.05 | 0.71 | - | - | 0.13 | 0.52 | - | - | -0.15 | 0.34 | - | - |  |
| **AF** | 0.36 | 0.008 | 0.24 | 0.03 | 0.52 | 0.005 | 0.31 | 0.21 | -0.48 | 0.006 | -0.25 | 0.20 |  |
| **ASD size** | 0.20 | 0.15 | - | - | 0.48 | 0.01 | 0.41 | 0.10 | -0.95 | <0.001 | -0.38 | 0.06 |  |

Delta indicates change of corresponding parameter between baseline and within 1year; HT, hypertension; AF, atrial fibrillation, LAVi (left atrial volume index).
